# Supplementary material for: Test of Gross Motor Development-3: Item Difficulty and Item Differential Functioning by Gender and Age with Rasch Analysis
Source: Int J Environ Res Public Health. 2022 Jul 16;19(14):8667. doi: 10.3390/ijerph19148667 (PMC9322710; doi:10.3390/ijerph19148667)
Supplement: Supplementary file 1 [file ijerph-19-08667-s001.zip › ijerph-1776967-supplementary.pdf]

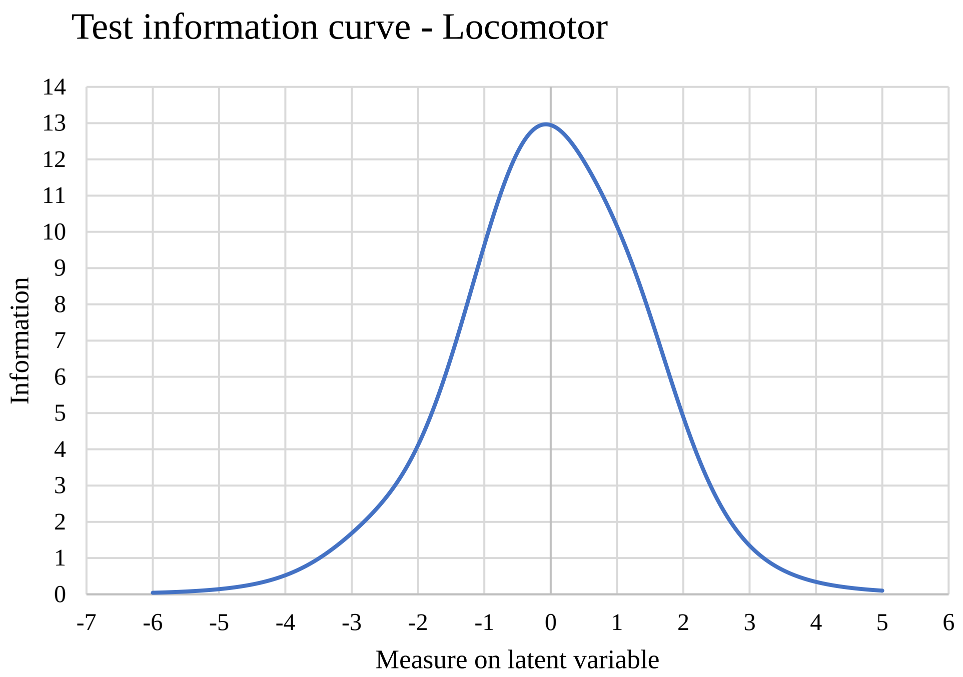

Figure S1. Test information function for locomotor dimension.

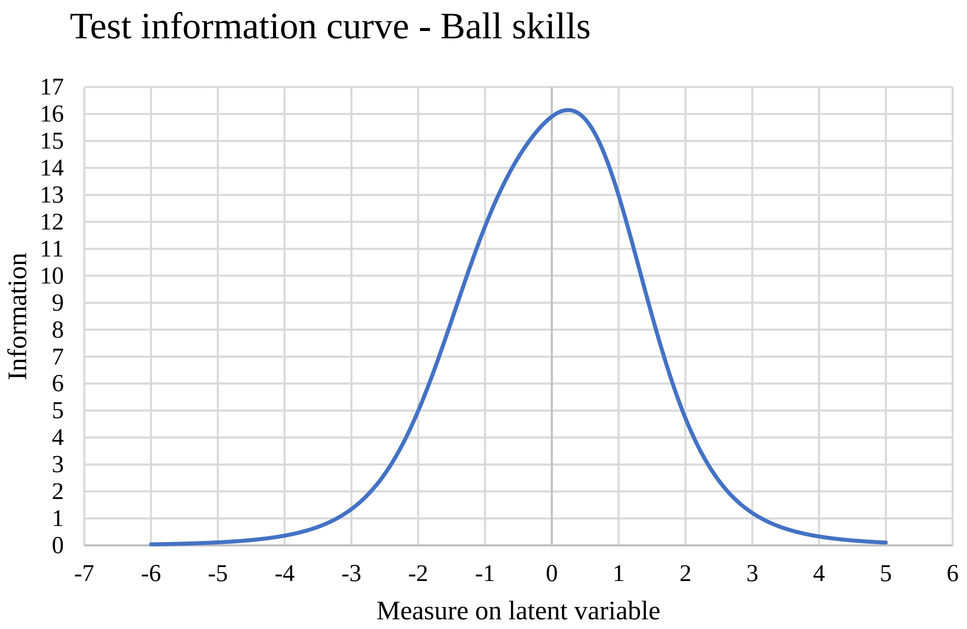

Figure S2. Test information function for Ball skills dimension.

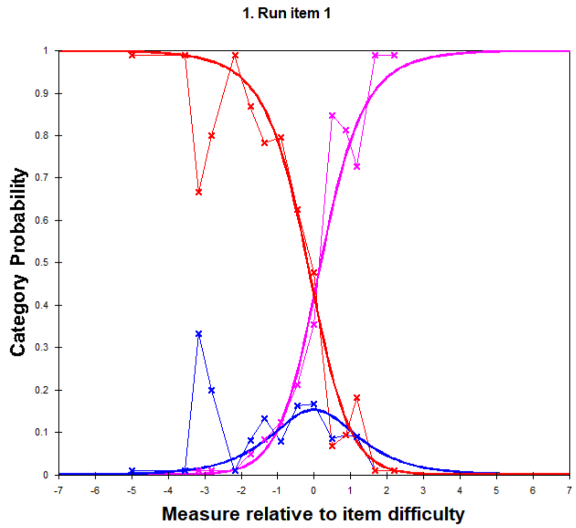
1. Run item 1
Category Probability
Measure relative to item difficulty

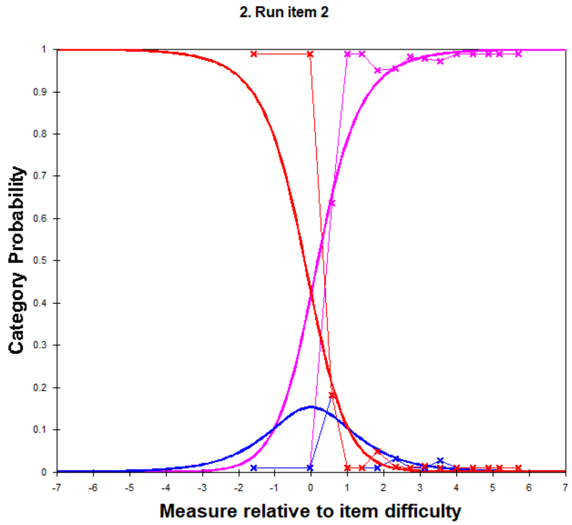
2. Run item 2
Category Probability
Measure relative to item difficulty

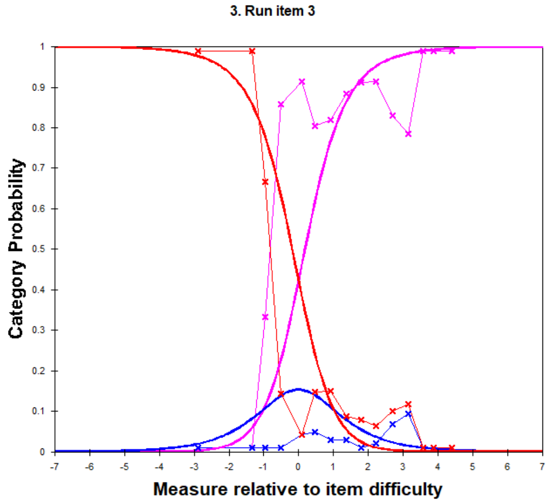
3. Run item 3
Category Probability
Measure relative to item difficulty

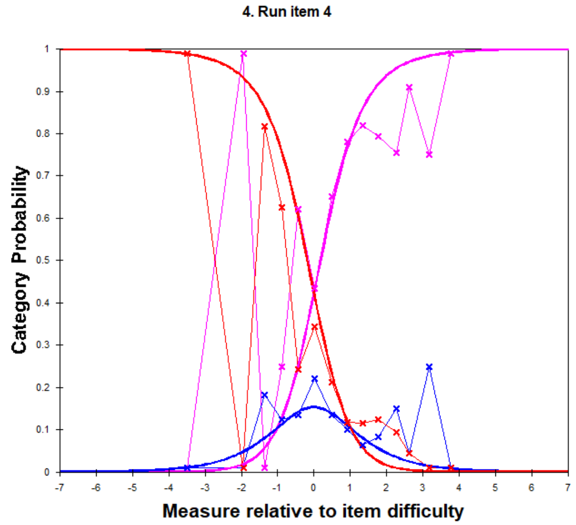
4. Run item 4
Category Probability
Measure relative to item difficulty

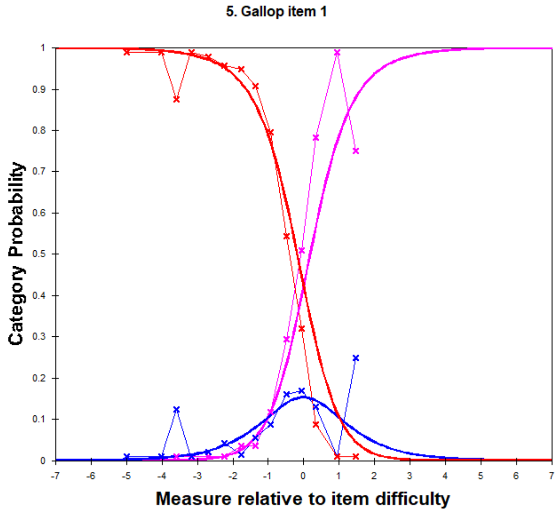
5. Gallop item 1
Category Probability
Measure relative to item difficulty

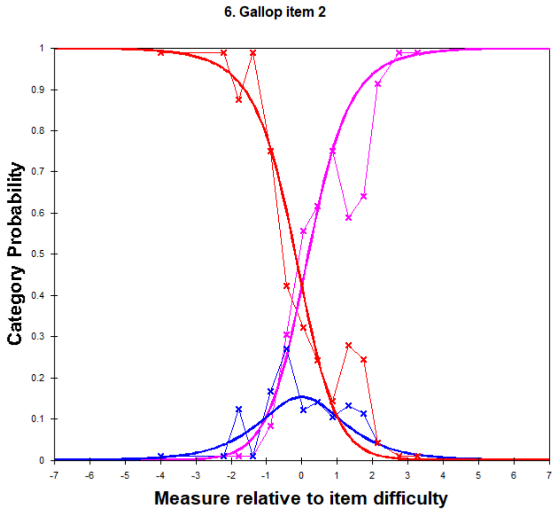
6. Gallop item 2
Category Probability
Measure relative to item difficulty

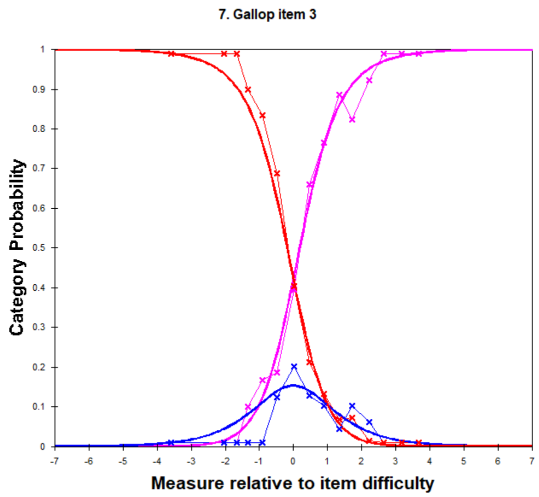
7. Gallop item 3
Category Probability
Measure relative to item difficulty

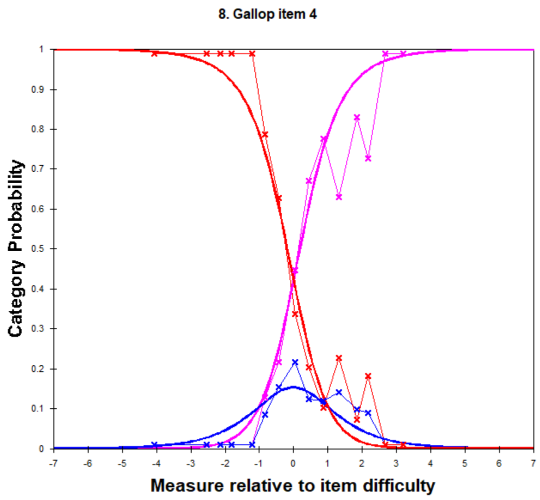
8. Gallop item 4
Category Probability
Measure relative to item difficulty

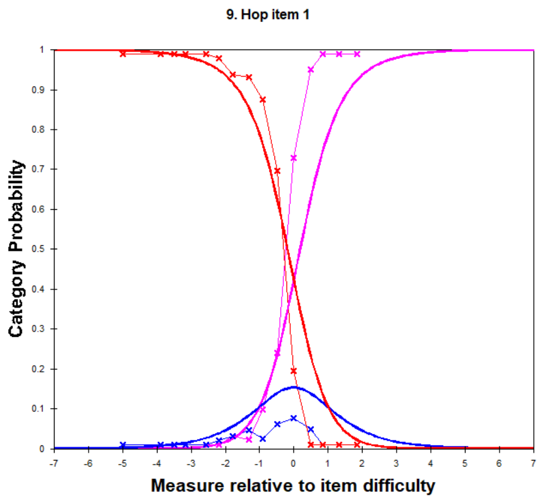
9. Hop item 1
Category Probability
Measure relative to item difficulty

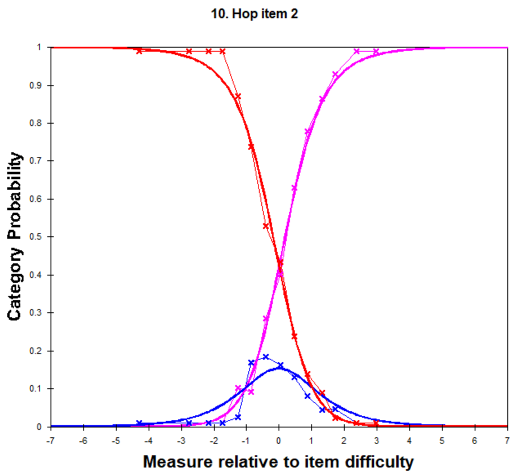
10. Hop item 2
Category Probability
Measure relative to item difficulty

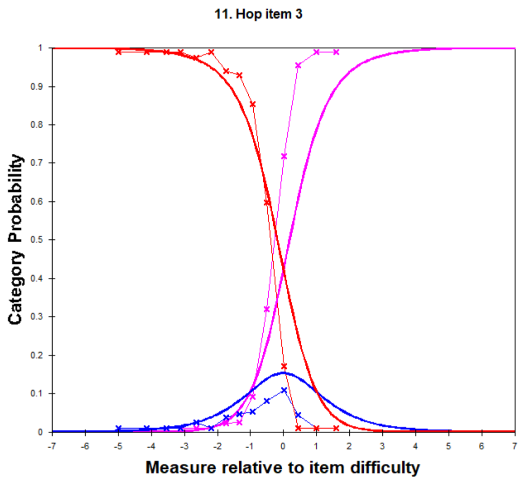
11. Hop item 3
Category Probability
Measure relative to item difficulty

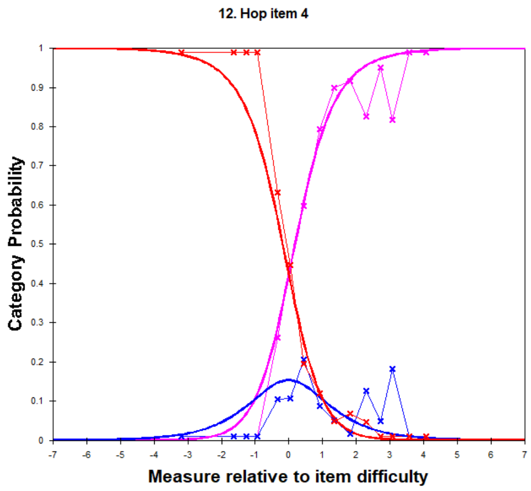
12. Hop item 4
Category Probability
Measure relative to item difficulty

13. Horizontal jump item 1

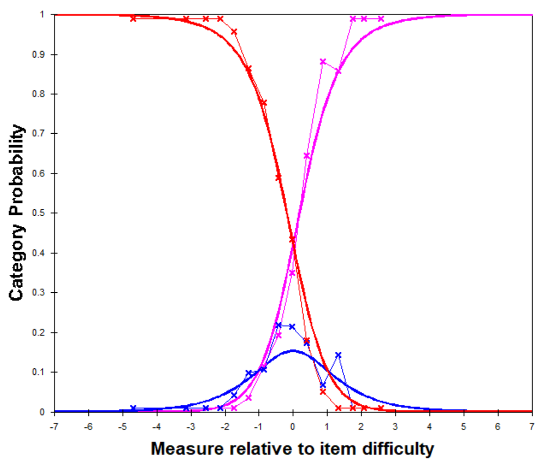

14. Horizontal jump item 2

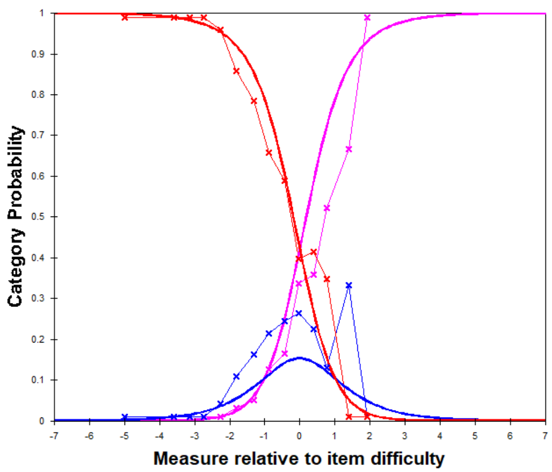

15. Horizontal jump item 3

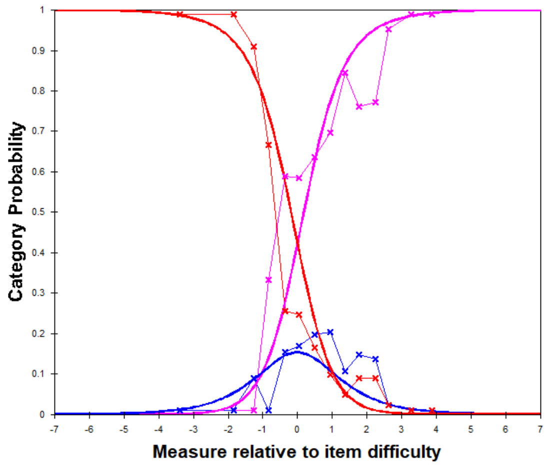

16. Horizontal jump item 4

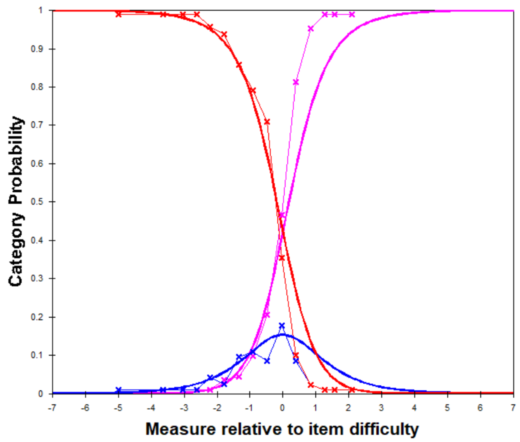

17. Slide item 1

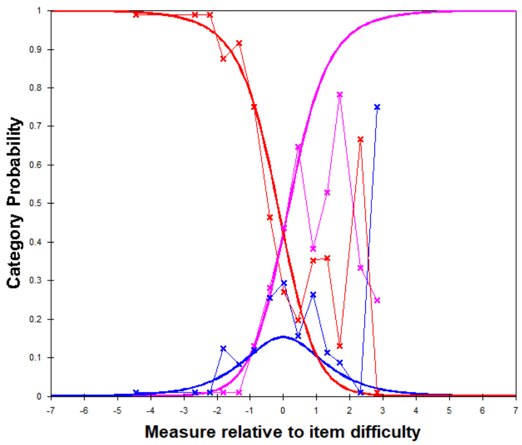

18. Slide item 2

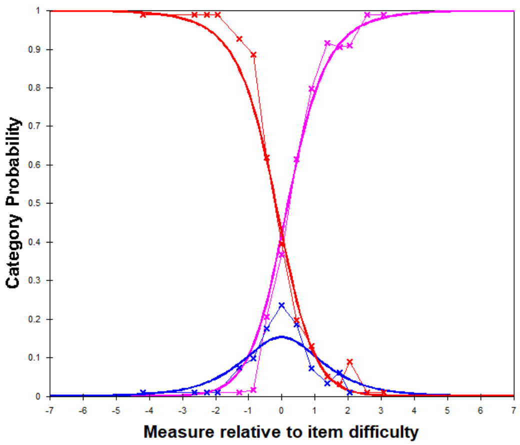

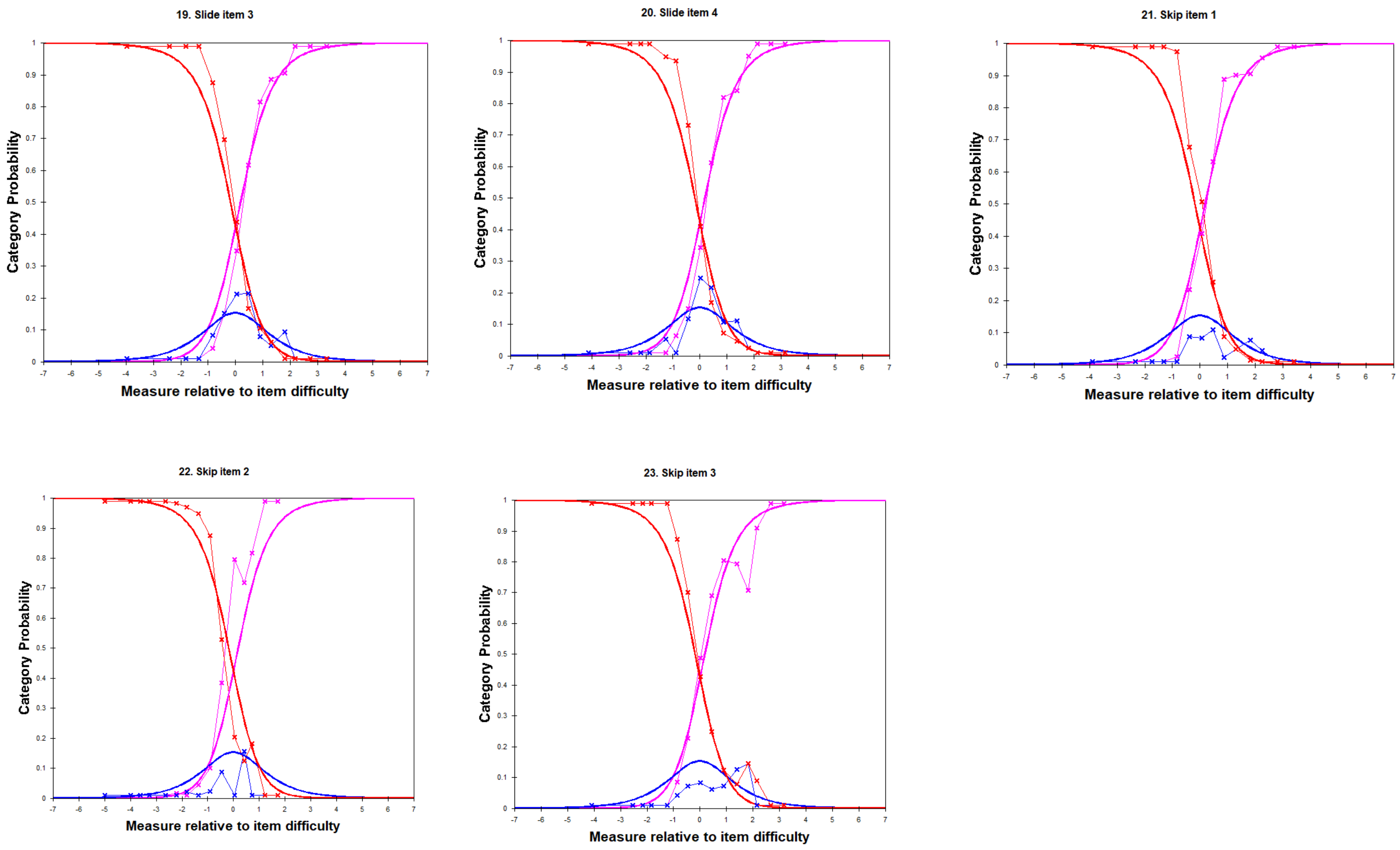

Figure S3. Measure relative to item difficulty for each item in locomotor skills.
